# From cells to pixels: A decision tree for designing bioimage analysis pipelines

**DOI:** 10.1111/jmi.70021

**Published:** 2025-08-29

**Authors:** Elnaz Fazeli, Robert Haase, Michael Doube, Kota Miura, David Legland

**Affiliations:** ^1^ Biomedicum Imaging Unit, Faculty of Medicine and HiLIFE University of Helsinki Helsinki Finland; ^2^ Data Science Center Leipzig University Leipzig Germany; ^3^ Center for Scalable Data Analytics and Artificial Intelligence (ScaDS.AI), Dresden Leipzig Germany; ^4^ Department of Infectious Diseases and Public Health City University of Hong Kong Kowloon Tong Hong Kong SAR China; ^5^ Bioimage Analysis & Research Okayama Japan; ^6^ Graduate School of Frontier Biosciences The University of Osaka Osaka Japan; ^7^ UR BIA INRAE Nantes France; ^8^ PROBE Research Infrastructure BIBS Facility INRAE Nantes France

**Keywords:** bioimage analysis, biological imaging, decision tree, image processing, microscopy

## Abstract

Bioimaging has transformed our understanding of biological processes, yet extracting meaningful information from complex datasets remains a challenge, particularly for biologists without computational expertise. This paper proposes a simple general approach, to help identify which image analysis methods could be relevant for a given image dataset. We first categorise structures commonly observed in bioimage data into different types related to image analysis domains. Based on these types, we provide a list of methods adapted to the quantification of images from each category. Our approach includes illustrative examples and a visual flowchart, to help researchers define analysis objectives clearly. By understanding the diversity of bioimage structures and linking them with appropriate analysis approaches, the framework empowers researchers to navigate bioimage datasets more efficiently. It also aims to foster a common language between researchers and analysts, thereby enhancing mutual understanding and facilitating effective communication.

## 1 INTRODUCTION

Bioimaging has revolutionised our understanding of biological processes. When studying these processes, the acquisition of images, for example using microscopy, is merely the first step. As microscopy techniques evolve and datasets become more complex, one crucial challenge is extracting meaningful information from bioimaging data. This requires defining analysis goals, exploring potential solutions, designing complete analysis workflows, and interpreting the data. These workflows require a diversity of skills for interconnecting the biological question, the specificities of the image acquisition device(s), the mathematical concepts of image analysis, and the knowledge of relevant and available software.

Imaging core facility staff are increasingly involved in the design of bioimage analysis workflows, and are often solicited for providing application or training of bioimage analysis. In response, many core facilities invest time and effort in educating staff on digital image analysis and training them with diverse software tools to provide more comprehensive help and support. Additionally, research groups may hire professional image analysts, mainly computer scientists, to assist biologists with bioimage analysis questions. Finally, the number of specialised core facilities dedicated to providing image analysis as a service has increased during the past few years.^1^, ^2^

The increasing interaction between biological and computational scientists emphasises the importance of establishing a common language between domain specialists and bioimage analysts. First, the scientific problem must be translated into an imaging experiment. Then, the resulting experimental data represent a bioimage analysis problem involving two types of experts. Domain specialists must be able to clearly articulate the scientific problems they seek to address through bioimage analysis. Conversely, analysts need to be able to process this information and provide results in a manner that is not only accurate and reliable but also interpretable by researchers in their respective fields. Having a common language also helps the analyst and biologist to assess when better quality data is required and possible, and to ensure they acquire the right data to address a given biological question. Therefore, fostering a shared language and bridging the gap between the world of image analysis and biological research is crucial.

A large number of efforts have been undertaken to bridge this gap. Several reviews have emphasised the importance of acquisition setup to be able to relate images to quantitative information,^3^, ^4^, ^5^, ^6^ including good practices for meta‐data.^7^ A large amount of literature was also devoted to the design of relevant image analysis workflows, including pre‐processing, segmentation, and analysis steps.^3^, ^4^, ^8^, ^9^, ^10^, ^11^, ^12^ Other reviews focused on the biological question or on the nature of the biological structure under investigation.^13^, ^14^, ^15^, ^16^ As the establishment of a common language was crucial to bridge people from different communities, ontologies have been developed and made available.^12^, ^17^, ^18^, ^19^ A large number of bioimage analysis software programs have been developed, making it necessary to classify and reference them.^20^, ^21^, ^22^, ^23^ Also, the Network of European BioImage Analysts (NEUBIAS) project gathered and organised some of these efforts, in training schools,^24^ bioimage analysis workflow‐centred books,^12^, ^18^ and the bioimage informatics index (BIII) database.^19^

Here, in addition to these numerous efforts, this manuscript proposes a guideline that first categorises structures commonly observed in bioimage data into different archetypes, and then identifies which image analysis methods could be relevant for each archetype. The categorisation of structures observed in bioimage data is related to image analysis vocabulary: ‘region’, ‘image texture’ or ‘point pattern’. To identify the category that can be associated with image data, we propose a visual decision tree that summarises the choices based on the visual features of the image rather than on the biological function of the observed structure. Each structure type is illustrated with a series of examples that associate a biological structure with an acquisition scale. Once categories are identified, we provide a list of methods adapted to the quantification of each category. Although some families of methods are more common in domains other than biology, such as material sciences or mathematics, we try to provide examples from biological studies. This way, the information is visually comprehensible and easily digestible, and ultimately facilitates the common understanding between researchers and bioimage analysts.^21^

While we will not discuss here the initial processing steps that are often necessary before undertaking bioimage analysis, it is important to highlight their importance. These steps are highly dependent on the datasets and may include several of the following steps: noise removal, registration, filtering, and segmentation methods, among other things. Segmentation by itself is one of the most important topics that any image analyst deals with and plays a vital role in preparing images for more advanced analysis. Many reviews have been published, some of them focused on microscopy image data.^16^, ^25^, ^26^, ^27^, ^28^ These steps are integral to ensure the accuracy, reliability, and reproducibility of the analysis results and, therefore, should not be overlooked. In addition, we do not specify specific software to use for each category as this is a preference of each analyst and depends on the size of the data in addition to the type and the resources available. Moreover, there have been other efforts in categorising and suggesting tools per category that are already available.^10^, ^21^, ^29^

With this in mind, we aim to provide some key concepts of bioimage analysis organised in a comprehensive way, to help the early career researchers to quickly identify which methods are the most relevant to their questions. This minimal visual chart of bioimage structures and analysis types is designed not just as a reference but also as a tool that empowers researchers to engage in effective communication with image analysts, ensuring mutual understanding. Furthermore, it will help researchers clearly define their image analysis objectives and comprehend the initial steps necessary to achieve their goals.

## 2 CHOOSING THE RELEVANT STRUCTURE TYPE

**FIGURE 1:**
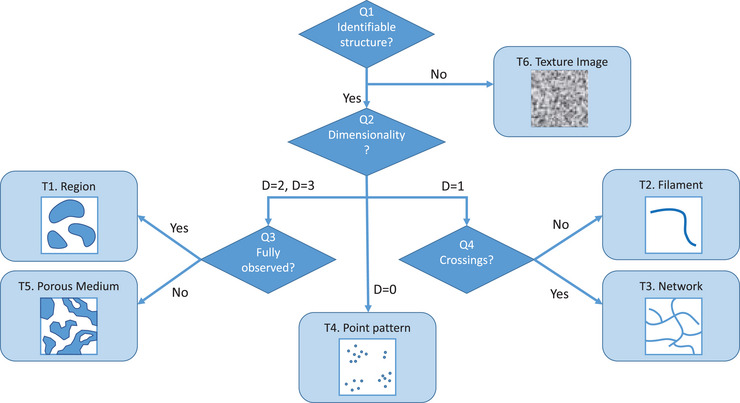
Decision tree for identifying the relevant structure type within the image.

This section classifies diverse structure types one might encounter in bioimaging. This classification is based mostly on the morphology of the structures, i.e. how they appear, rather than on what they are. We propose the decision tree presented in Figure 1 to identify the structure type that best fits the content of the image. This decision tree is based on a series of questions (identified by Q1 to Q4), leading to different structure types (identified by T1 to T6). The different choices and categories are detailed hereafter. Once the structure type is identified, different families of image analysis methods may be envisioned; they are presented in the next section. Table 1 also provides a list of short definitions for technical terms that are used through this article.

**TABLE 1:** Glossary of key technical terms in image analysis.

**Bioimage analysis**	Bioimage analysis is a process of identifying spatial and temporal distribution or dynamics of biological components in image data and measuring their characteristics to study their underlying mechanisms in an unbiased way.
**Bioimaging**	The acquisition of images, typically using a microscope, in a biological context.
**Boundary**	The points within the image that are located at the interface of two specific regions, for example, separating a cell from the background. Boundaries may be manually delineated, or obtained from a binary image of the region.
**Connected component**	A region with the property that each pair of points within the region can be connected by a path totally included within the region. If not, the region is composed of several **disjoint** parts.
**Filament (curve, line)**	A structure that can be assimilated to a single curve in the plane or in the space. Several filaments can form a network.
**Identification**	Identification is the process of associating a class or a label to a region or a position within an image.
**Medial axis** **(or skeleton)**	The medial axis of a region is the set of points within the region having more than one closest point to the boundary of the region.
**Network**	A structure composed of several filaments that can interconnect, cross, or divide.
**Porous medium**	A structure of interest with a complex morphology that is difficult to consider as a single region or collection of regions and that may be not fully observed within the field of view of the image.
**Region**	A portion of an image that can be manually delineated by an expert. A region of interest often corresponds to the notion of region. Within this manuscript, it also encompasses point patterns, filaments, or networks that are not considered within region analysis.
**Segmentation**	The segmentation of an image can be defined as the (manual or automated) partition of the image into distinct regions.
**Skeletonisation**	The image processing operation that consists in computing the medial axis, or skeleton of a region. Skeletons may be analysed as networks, or simplified to filaments.
**Structure of Interest**	A subset of the sample under investigation.
**(Image) texture**	Image texture can be defined as the local variations of grey levels within the image.

### 2.1 Q1: Can you identify a structure?

**FIGURE 2:**
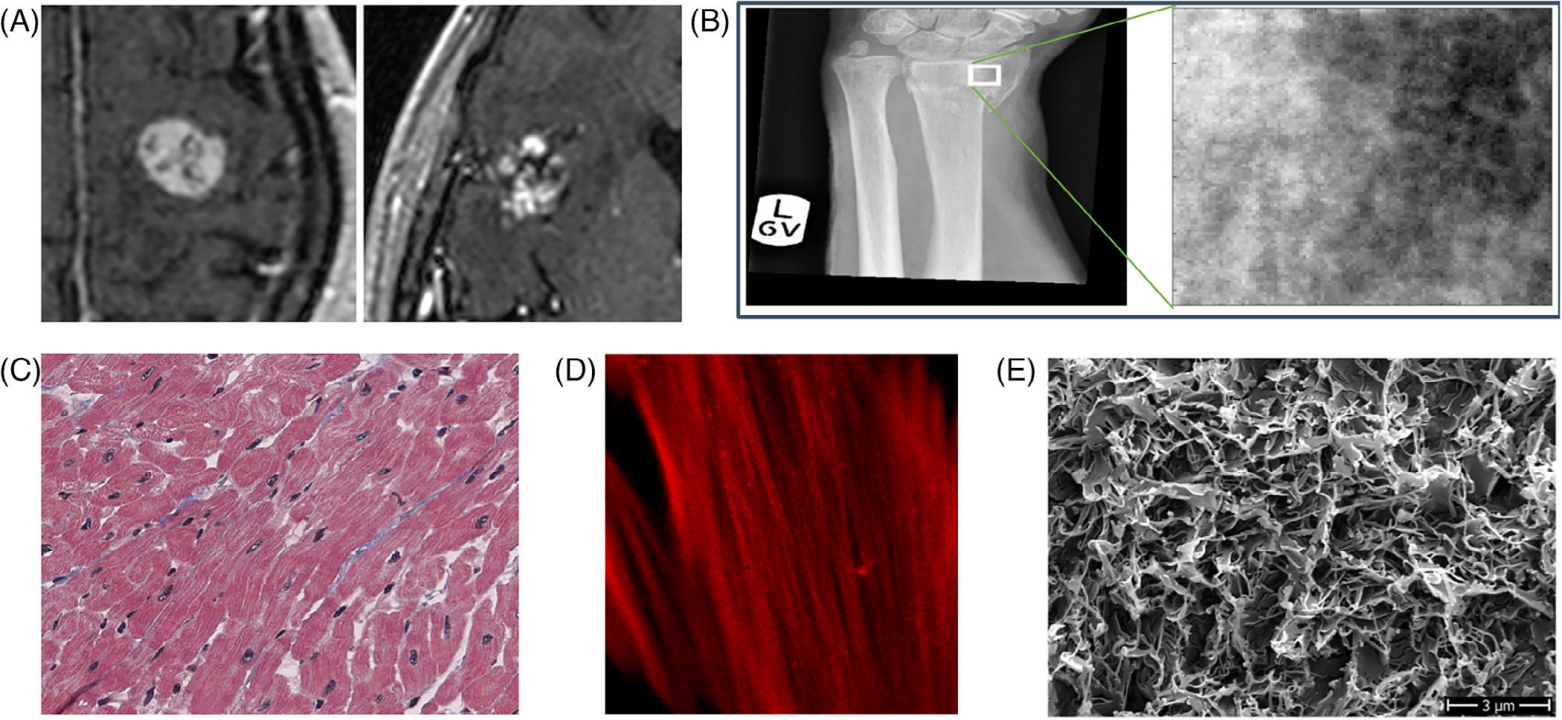
Examples of images illustrating the notion of image texture. (A) Discrimination of brain tumours within MRI images (from Larroza et al.^30^). (B) Study of wrist fractures from X‐ray radiography images (from Reyes‐Aldasoro et al.^31^). (C) Heart tissue slice imaged with high‐resolution slide scanner (from Zach et al.^32^). (D) Cytoplasmic actin network (from Luo et al.^33^). (E) Changes of microstructure of grape cuticle observed with electron microscopy (from Herzog et al.^34^), scale bar 3 µm.

The first question to consider is if we can identify a structure of interest within the image. The structure of interest often corresponds to a biological structure, such as a cell, an organelle, an organ, a tissue, etc. It may also correspond to a region where the pixels appear different from the surroundings, such as a lesion or a tumour within a tissue. In some cases, it may not be obvious how to identify the structures of interest within images. The reasons may be that the resolution or the contrast is too low to accurately identify the structure, or that the structure is difficult to determine uniquely from its contrast. In such cases, instead of forcibly delineating the outline of a hardly definable region, an alternative is to quantify the local variations of grey levels within neighbour pixels simply by measuring the intensity profiles crossing the region where one needs to examine the changes in intensity in detail. Furthermore, content‐rich measurements can be achieved, by applying image texture analysis methods (See Figure 2).

Image texture analysis quantifies the local variations of grey levels within the image. This family of methods has proven powerful in medical imaging to describe changes between tissues or regions of interest when the differences come mostly from fluctuations in the signal rather than from morphology or global intensity. Typical examples include the detection of tumours from changes in texture features computed from MRI data,^30^ X‐ray radiography images,^31^, ^35^ or ultrasound images.^36^

Examples of texture analysis:

- Tissue sections: Evaluating different tissue types, especially in histology, where textural differences might hint at pathological changes or disease states.^32^ Additionally, in the field of cancer research identifying malignant from benign tumours based on texture irregularities, aids in cancer diagnosis and treatment studies.^30^
- Cell colonies: Differentiating between varying cell colonies in a Petri dish based on their growth patterns.^37^
- Plant leaves: Classification of plant leaves achieved by using texture features.^38^
- Patterns of collagen and elastin within the extracellular matrix, observed with a resolution making it difficult to identify individual bundles.^39^, ^40^
- Patterns of bundled actin filaments observed at a scale making them difficult to individualise.^33^

One criterion to decide whether the content of the image presents a structure could be: Can we manually delineate a region of interest? Another criterion could be: Can it be binarised? If yes, we can consider Q2. Otherwise image texture analysis can be envisioned.

### 2.2 Q2: What is the structure's dimensionality?

If the structure is identifiable (Q1), another criterion to identify its type is to consider its ‘dimensionality’. Intuitively, the dimensionality can be related to the number of directions a point can go within the structure. In most cases, one considers ‘thick’ structures, or regions, that delineate an interior and an exterior. The dimensionality is the same as the one of the image (two for planar images, three for three‐dimensional images). In these cases, continue with Q3.

It is also possible to consider elongated structures with very small thickness, such as curves, filaments, or an interconnection of several curve elements. In these cases, the dimensionality equals one, as a point within a curve can move only in one direction along the curve. Note that in the case of regions with narrow thickness, it is possible to focus on its medial axis, or skeleton, and perform analysis on this curve shape. If the structure of interest has the dimension of a curve, continue with Q4.

A last case occurs when all the dimensions of the objects become negligible or irrelevant, and objects can be reduced to points corresponding to their position. Instead of regions, observations correspond to a collection of punctual observations, also referred to as a point pattern. Examples of point patterns in bioimaging include some organelles within a cell, for example, vesicles, or proteins appearing as fluorescent dots. Point patterns may also be derived from more abstract constructions such as the centroids of well‐defined regions such as cells or nuclei. Note that in some cases, approximation of structure as points may over‐simplify biological events as it discards too many details. The analyst should carefully consider if the goal of the analysis matches with this approximation by asking ‘can we really analyse this structure as a point pattern?’

When considering dynamically moving biological structures such as intracellular vesicles, chromosomes, cells within tissue, these structures are analysed by tracking their changes in positions. Often, the position of these structures at each time point is represented by a single point, and their movement is then measured by time‐sequence of the displacement of these points.

**FIGURE 3:**
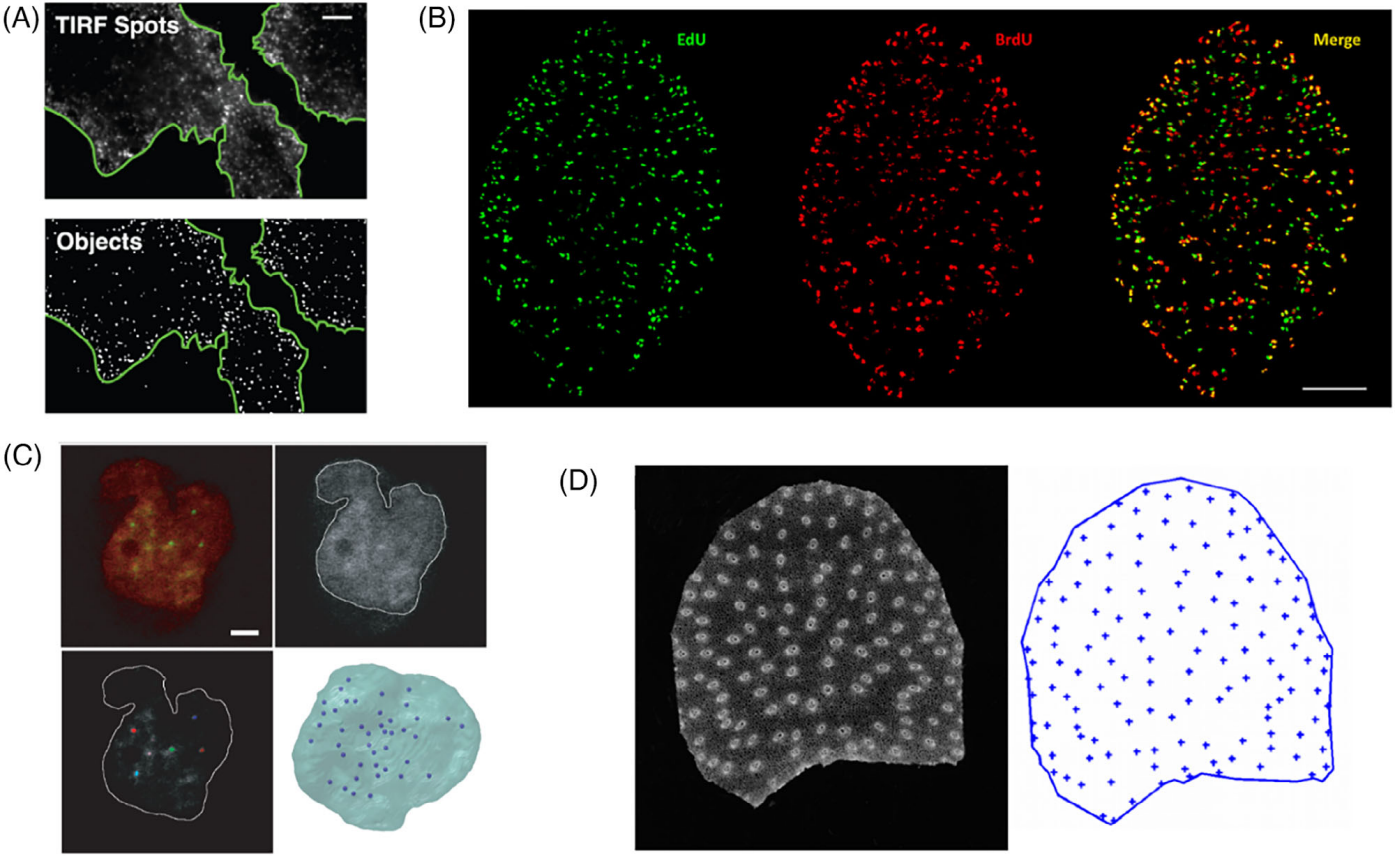
Examples of structures that can be interpreted as point patterns. (A) Quantitative analysis of spatial endocytic sites,^49^ scale bar: 5 µm. (B) Dual labelling of thymidine analogies within *Fasciola hepatica*,^43^ scale bar: 100 µm. (C) Analysis of 3D spatial organisation of centromeres within nucleus,^45^ scale bar: 2 µm. (D) Identification of the positions of vascular bundles within a maize stem cross‐section.^48^

Examples of point patterns:

- Synaptic vesicles/puncta: In neuronal studies, the distribution of these vesicles can offer insights into synaptic strength, plasticity, and changes associated with various conditions or treatments.^41^
- Cell membrane receptors: The clustering or distribution of specific receptors on a cell surface can hint at cellular signalling potential and sensitivity to external cues.^42^
- Protein complexes: In cell biology, observing the spatial arrangement of certain protein complexes can shed light on cellular processes, for example, endocytic machinery^43^, ^44^ (Figure 3B).
- The spatial organisation of chromocentres within a nucleus can be described by point pattern^45^ (Figure 3C).
- Single molecules resolved by Single Molecule Localisation Microscopy (SMLM) exhibit point patterns and are treated as point clouds to render their clusters and structures.^46^
- Within tissues, the centroids of cells, or the localiation of cell junctions generates a point pattern^47^ (Figure 3C).
- The localisation of small organs or tissue within an enclosing organ or organism can be described through point patterns^48^ (Figure 3D).

### 2.3 Q3: Is the structure fully contained within the field of view?

**FIGURE 4:**
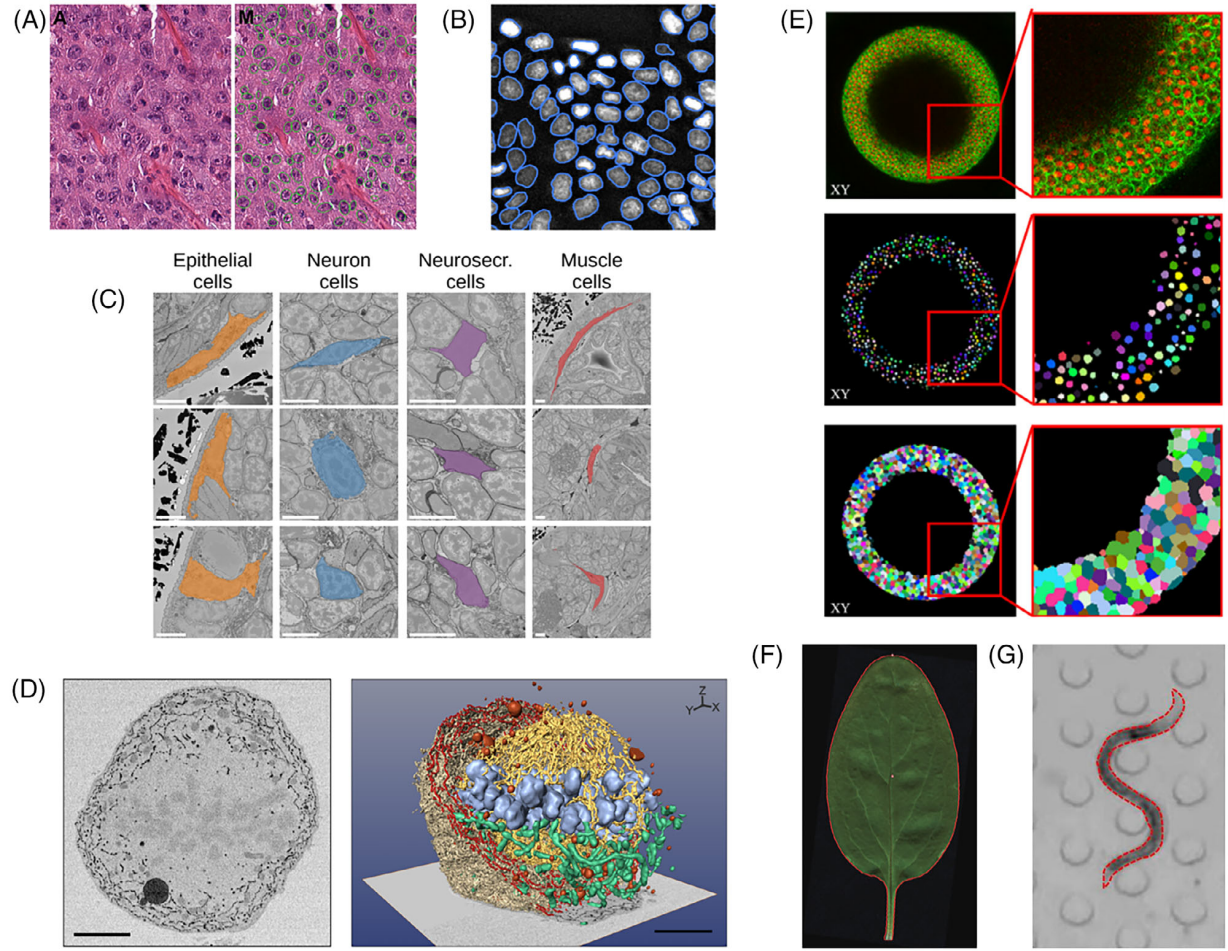
Examples of regions in bioimaging studies. (A) Automated segmentation of breast cancer nuclei from H&E‐stained tissue section (from Veta et al.^50^). (B) Segmentation of nuclei outlines from fluorescence microscopy images (from Englbrecht et al.^51^). (C) Cells from distinct tissues have been segmented on electron microscopy images (from Zinchenko et al.^52^), scale bars: 5 µm. (D) Segmentation of various organelles within a cell from TEM images (from Belevich et al.^53^), scale bars: 5 µm. (E) Joint segmentation of cells and nuclei in synthetic 3D data structures (from Eschweiler et al.^54^). (F) Segmentation of a leaf from a digital camera image. (G) Segmentation of a nematode to study its motility (from Sznitman et al.^55^).

In many cases, the biological question leads us to consider one or several distinct regions within an image. These regions often correspond to a specific biological structure, such as cellular organelle, for example, nuclei (Figure 4B), cells (Figure 4A, B, and E), at a larger scale, organs (liver, kidney, plant stem or leaf) (Figure 4C), or whole organisms (Figure 4D and F).

From an image analysis point of view, a region is a set of pixels or voxels that share common properties, such as the same range of intensity or of colour, an area or volume separated by continuous boundary, or the same location with respect to a reference structure. Technically, the region is often assumed to be composed of a single ‘connected component’.

Examples:

- Nuclei as observed in brightfield or confocal microscopy^56^ (Figure 4B).
- Tissue culture cells observed in brightfield imaging.^57^
- Lysosomes, often defined as spherical vesicles^58^ (Figure 4D).
- Some organs in the human or animal body: liver, kidney, heart, etc.^59^, ^60^
- Grains, leaves, fruits, or other plant organs observed with camera imaging.^38^, ^61^, ^62^
- Individual animals observed with camera or video acquisitions to track their behaviour.^55^, ^63^, ^64^

**FIGURE 5:**
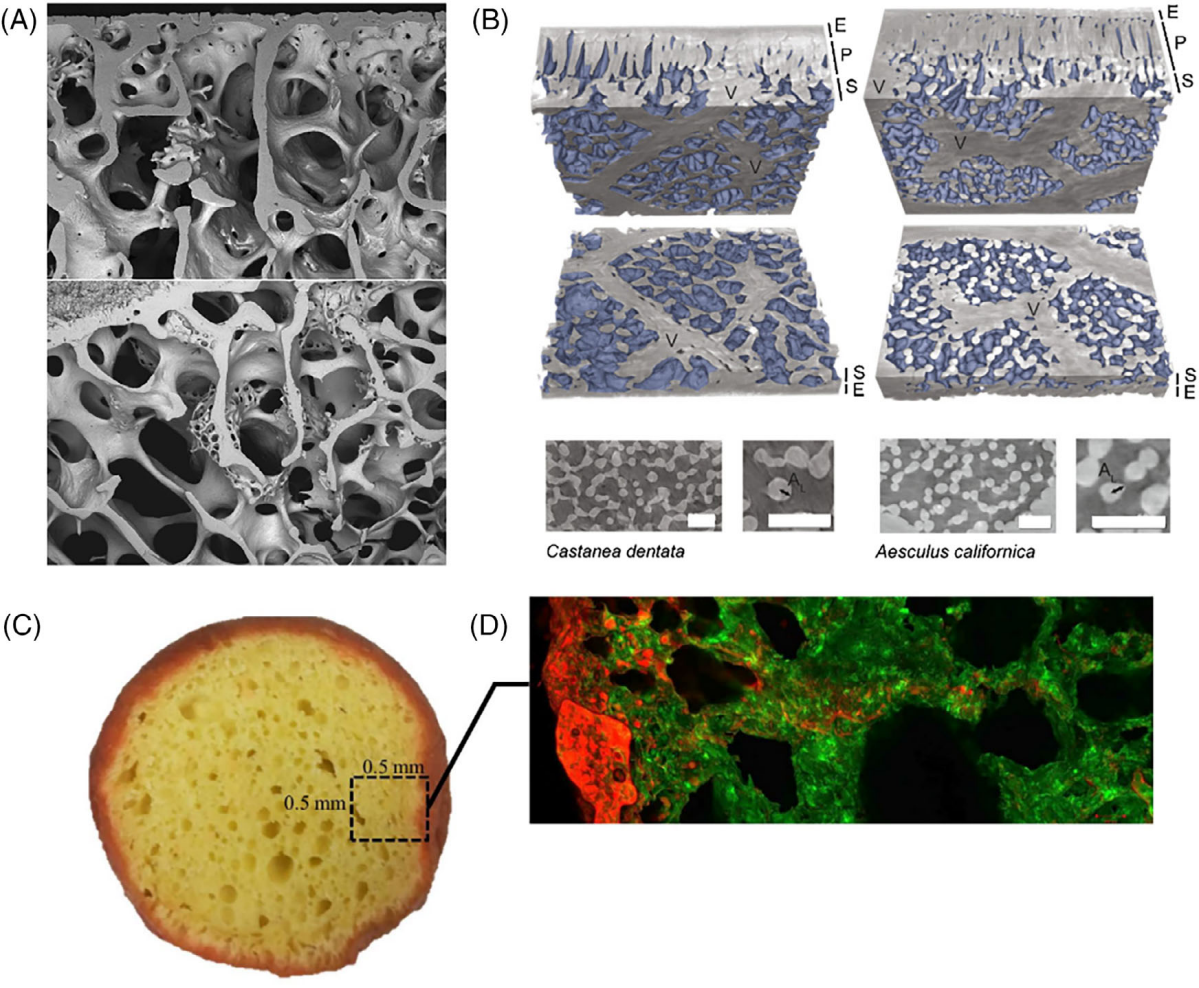
Images of biological structures that can be interpreted as porous media. Trabecular bone (A) is an open‐celled foam composed mostly of collagen, hydroxyapatite mineral, and osteocytes. Here the intervening soft marrow tissue has been removed and the dry bone tissue imaged in scanning electron microscopy.^66^ (B) Spongy plant mesophyll has a porous structure. X‐ray microtomography images on two different species show the epidermis (E), palisade mesophyll (P), spongy mesophyll (S), veins (V), and intercellular airspace (shaded blue)^73^ scale bars 50 µm. A photograph (C) and confocal scanning light microscopy image (D, red = oil, green = batter) demonstrate the porous structure and oil penetration depth in magwinya, a type of fried dough popular in South Africa and Botswana.^78^ All images reused under CC‐BY terms.

Some biological structures such as portions of bone imaged at microscopic scale, or lung alveoli, present a ‘sponge‐like’ structure with a complex morphology that are not readily described with classical region features.^65^ In particular, the structure is often observed within a limited field of view, and/or it is made up of a large number of elements that interconnect in a complex 3D architecture with ramifications and/or void networks. This structure type is commonly encountered in material sciences, where the term porous media may be encountered. Various examples of porous media may be however considered in biology (see also Figure 5):

- Bone microstructure: offers insights into bone strength and health^66^, ^67^, ^68^ (Figure 5A).
- Lung tissue: interconnection between air and blood, with a large number of collapsing alveoli.^69^, ^70^
- The morphology of medical implants or scaffolds seems, in particular the porosity, strongly affect their biocompatibility.^71^, ^72^
- The morphology of plant tissues can be analysed as a porous medium^73^, ^74^, ^75^ (Figure 5B).
- Foods products such as bread and cereal products^76^, ^77^, ^78^ (Figure 5C), or ice cream.^79^
- Soils, which are important habitats for plant roots and microorganisms such as protozoa, bacteria and fungi.^80^

### 2.4 Q4: Do curves have crossings?

Many biological structures such as vessels, plant roots, or membranes appear as thin objects, looking like a curve (or filament) with a constant thickness range, or a superposition of many curves. With such structures, one can approximate them as curves, neglecting the fact that they do have some area or volume, as long as the focus of the research interest allows this ‘simplification’. For this approximation, one may consider either a main axis of the structure, such as the medial axis of a dendrite, of a microtubule, or of the root of a plant.

Examples of filament‐like structures in bioimaging:

- Axonal structures within white matter.^81^
- Xylem vessels within wood,^82^ or more generally vascular bundles within plants.^48^
- Filopodia.^83^, ^84^
- Microtubules of plant cells, for example, cortical microtubules.^85^
- Microtubules of animal cells.^86^
- Stress fibres in cultured cells.^87^

Note that in the case of 3D imaging it is possible to consider surfaces as two‐dimensional structures embedded within a three‐dimensional space. Within this manuscript, this case integrated into the ‘filament’ structure type.

Curve structures may present a large number of crossings and/or branches. In these cases, it is more appropriate to consider them as networks, composed of a large number of elementary curves meeting at junction points. In terms of image acquisition, it may be necessary to acquire tile scans over larger areas to capture the entire network. In such cases, one is more interested in the interconnection pattern of the network than the lengths and curvature of curved elements. From an image analyst's point of view, networks are typically considered as a (mathematical) graph structure that can be characterised by a variety of metrics including branching, connectivity density, or node degree distribution. The details of this topic are covered in the ‘networks morphology’ section.

**FIGURE 6:**
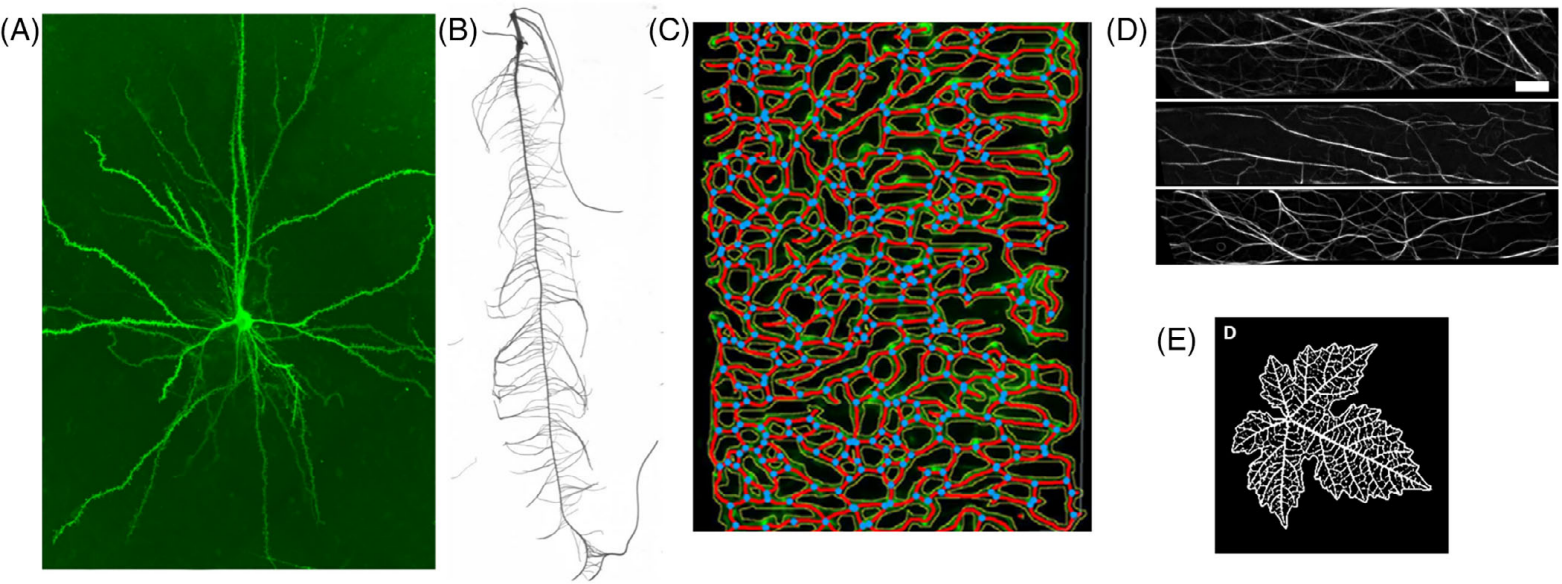
Examples of image structures representing networks, or collections of curvilinear regions. (A) Dendritic network of a neuron.^88^ (B) root network of maize (from Pace et al.^89^). (C) Angiogenesis in the embryonic hindbrain (from Zudaire et al.^90^). (D) Actin network in plants (from Hembrow et al.^100^), scale bar: 5 µm). (E) Venation network in a grapevine leaf (from Benfenati et al.^61^).

Examples of networks:

- Neurons: Studying neuronal networks, their branching and synapse formation gives insights into neuronal development, plasticity, and various neurological conditions^88^ (Figure 6A).
- Plant roots: the architecture of these structures serves critical functions including water and nutrient uptake^89^ (Figure 6B).
- Blood vessels: studying vascular network, with its arteries, veins and capillaries, assists in understanding angiogenesis, vascular diseases, and tissue perfusion^90^ (Figure 6C).
- Ophthalmology: retinal vessel network from fundus screening.^91^, ^92^
- Cell cytoskeleton^15^, ^93^, ^94^ (Figure 6D).
- Mycelium: In fungi, these structures are complex underground networks involved in nutrient absorption.^95^, ^96^
- Endoplasmic reticulum observed in 2D images.^97^, ^98^
- Pattern of the extracellular matrix observed on histological tissue sections.^40^
- Vasculature network within plant tissues^99^ (Figure 6E).

## 3 HOW TO IDENTIFY THE RIGHT STRUCTURE CATEGORY

Identification of the appropriate category for image analysis is not always a simple task. For example, depending on the type and the magnification of the acquisition device, neurons can be seen either as nearly convex regions (when considering the cell body), as a very ramified structure (when considering the surrounding dendrites), as a network of dendritic connections (when considering a population of neighbour neurons), or as a point pattern (when considering a slice of neuronal tissue). Moreover, ‘what they look like’ can be different after going through image processing of original raw image data. For example, the cell body of a neuron can be converted to a point by centroid detection. Therefore, the name of the target biological structure (e.g., neuron) is not usually the key to finding the right category of image analysis, and rather the shape of the target of analysis itself is the primary key for identifying the image analysis category. When a researcher becomes experienced and knowledgeable on the parameters that can be measured for each image analysis category, it allows the researcher to design the required image processing steps to make the raw image data more amenable to a relevant image analysis method.

**FIGURE 7:**
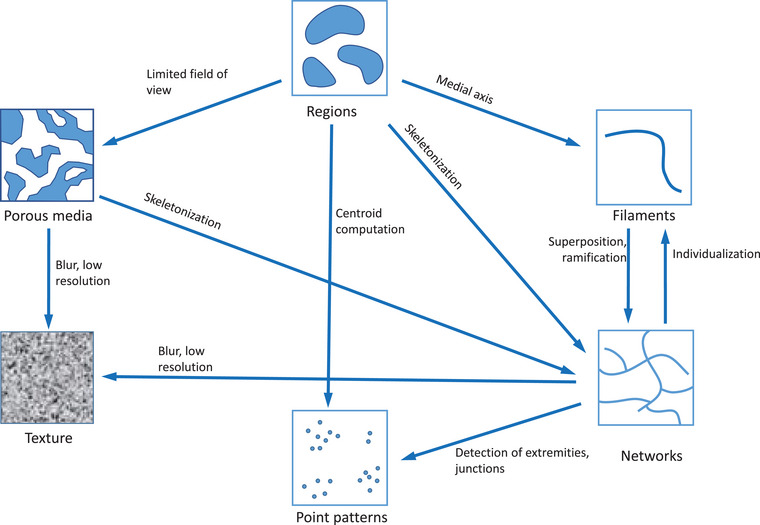
Overview of the different structure types that can be considered for setting up an image analysis workflow, and the relationships between them. Dashed arrows correspond to conceptual change, while solid arrows correspond to changes that can be obtained by image processing algorithms. Regions (left blue panel) can exhibit various morphologies: compact, small and numerous, elongated, and complex. When regions are minute, they can be represented by the centroid of each region and considered as point patterns. When regions are elongated, they may be assimilated into a single curve (right blue panel). Thin regions with very complex shapes can be considered as a network structure (right blue panel). Complex regions with a limited field of view may be better described as porous media.

These different categories of image analysis and the relationship between them are illustrated in Figure 7. The ‘classical’ case of image analysis considers regions that may present a large variety of morphologies: compact, elongated, composed of many small elements, complex, and ramified (Figure 7, left blue panel ‘Regions’). Depending on these morphologies, the simplification of shapes by image processing allows us to employ other families of image analysis methods. For example, assimilating small regions to points drives to point pattern analysis (Figure 7, top right ‘point patterns’). Extracting the medial axis of elongated regions orients towards filament analysis methods (Figure 7, right blue panel ‘Curves’). If the morphology of the region is very complex, it could be more appropriate to consider it as a porous medium (Figure 7, bottom right ‘Porous media’), or to compute its skeleton to consider network analysis of the skeleton (Figure 7, right blue panel ‘Curves’). The distinction between filament and network is sometimes difficult to do, and may depend on how much we can identify individual filaments from the network. Porous media and network analysis often assume that the structure can be well delimited from images. If this is not the case, texture analysis may be more appropriate. Overall, this categorisation may not be exhaustive, some categories may be further subdivided, and some studies may not fit within one of these categories. However, we believe that the proposed flow chart is helpful in many biological studies to identify appropriate image analysis methods based on observed patterns, the decisions of which are not obvious for the image analysis beginner.

The difficulty in choosing the right category may become partially solved by keeping in mind that a specific type that a biologist chooses for a biological structure is a ‘Hypothesis’. Rather than expecting that there is an intrinsic unique category each biological structure belongs to, each researcher hypothesised that that biological structure belongs to a category. This reasoning is similar to a modelling approach. For example, when investigating single cells with a sufficient resolution, we can hypothesise that each cell occupies a certain area. The cell is associated with an ‘area region model’, in the sense we want to measure certain properties observable within the cell. Alternatively, we can hypothesise that a single cell is ‘a point’, for example, the centroid of the cell area, if we want to measure the position of that cell. In this case, considering a ‘point model’ for cells is more appropriate. Hence, it is important to keep in mind that how we categorise biological structures depends firstly on how that shape visually appears, which is dependent on the magnification and the optics we use for imaging, and secondly on the goal of the measurement we want to achieve. For this second point, knowledge of what we can do with digital image processing and analysis is important, which can be used to choose the optimal hypothesis—or a shape category. This knowledge may also lead to the optimal choice of imaging modalities towards a desired measurement goal. In this way, the difficulty of choosing an image analysis for randomly taken image data becomes diminished. For such an image analysis‐dominant imaging strategy, knowledge of what can be analysed for each structure type is crucial, and we explain this in the following section.

## 4 DIFFERENT ANALYSIS APPROACHES

**FIGURE 8:**
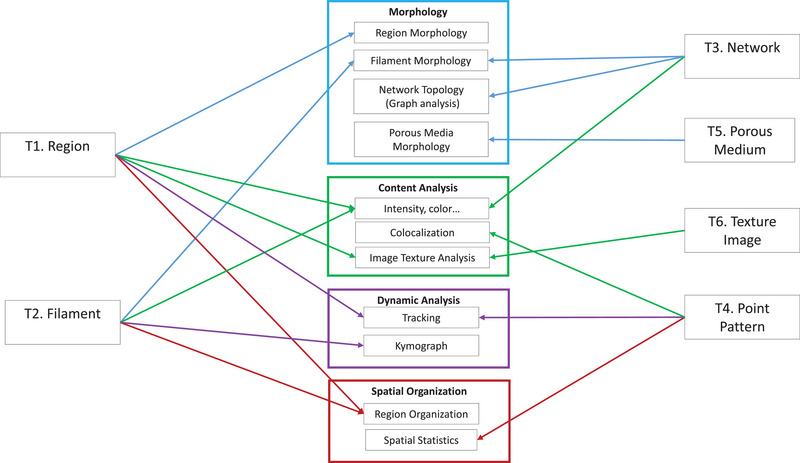
Identification of the families of image analysis methods that can be considered for each type of structure.

The choice of the most relevant image analysis method strongly relies on the type of structure that can be identified from the image. In addition, image analysis features may be classified according to the nature of the information they describe. Within this manuscript, we organise features into four categories: morphology, content (or pixel values), dynamic (taking into account the temporal variations within images), and spatial organisation. Depending on the structure type, image analysis categories have to be considered from a different way. For example, the morphology of a compact region is not described the same way as the morphology of a network‐like structure. Figure 8 presents a summarised view of a selection of features that can be quantified for each image structure, organised into the categories described hereafter.

## 5 MORPHOLOGY ANALYSIS

### 5.1 Regions morphology

A typical way to describe regions is to quantify their morphology, by computing morphometric features that describe their size and shape.^4^, ^101^, ^102^, ^103^, ^104^

For regions retrieved from two‐dimensional images, typical size features comprise the area and the perimeter. The size is often investigated through the spatial extent of the region, which can be evaluated by several features (Feret diameter, enclosing disc diameter, etc.). Morphology features may be computed from a derived region such as the convex hull, the bounding box or the enclosing disc. The computation of an equivalent ellipse that presents the same statistical moments is also of common use, by providing an intuitive evaluation of the position, orientation, and dimensions along the main directions. Counterparts are often clearly defined for three‐dimensional regions (volume, surface area, equivalent ellipsoid, etc.). The ‘width’ or the ‘thickness’ may also be related to the size, while their definitions are less consensual: diameter of largest inscribed disk, width of the oriented bounding box, average thickness computed over a medial axis within the region, etc. For this reason, the use of the ‘width’ or the ‘thickness’ features requires clear definitions provided whenever used for analysis.

It is often desirable to quantify morphology of regions independently of their position, size, or orientation. Accordingly, within the domain of shape analysis, a large number of shape features have been defined in order to be invariant with geometric transformations composed solely of translations, rotations, and scalings. Such features are most of the time based on (normalised) ratios of size features. Typical examples comprise elongation of equivalent ellipse, circularity, roundness, convexity, etc.^3^, ^4^, ^103^ A visual overview was recently presented in table 2 of Culley et al.^4^ For three‐dimensional regions, similar shape indices may be defined as well.^45^, ^67^, ^105^ More complex shape features may be obtained by considering a parameterisation of the contour and computing the Fourier transform of the coordinates, leading to Elliptic Fourier Descriptors.^62^, ^102^, ^106^, ^107^ Such descriptors provide a more detailed characterisation of the individual regions, making it more efficient to apply machine learning methods for clustering or discriminative approaches. The boundary of three‐dimensional regions can be analysed in a similar way using spherical harmonics.^108^, ^109^, ^110^

In anatomy, physiology, or developmental biology, regions may correspond to well‐known structures (bones, skulls, specific organs, etc.). They may have highly complex morphologies that are difficult to describe with a few summary features. A common solution consists in identifying a set of landmarks that correspond to characteristic points, and quantifying the shape by computing the ratios of distances between pairs of landmarks.^111^, ^112^, ^113^ This type of methodology, referred to as ‘allometry’, is often involved in evolution and development studies.

Once a number of features have been quantified within a collection of regions, classical statistical descriptions, and testing allow us to detect differences in phenotypes with individual feature values. If the number of features is large, the combination of dimension reduction and unsupervised learning (clustering) allows the identification of populations of regions that share similar features.^52^, ^107^, ^114^, ^115^ Another option is to build a machine learning classifier that will identify which group each region belongs to Kalinin et al.,^116^ Pieczywek and Zdunek,^117^ and Wiggins et al.^118^

Besides the extraction of features, the computational anatomy formalism approach considers describing populations of shapes by projecting them into an abstract ‘shape space’, obtained by computing geometric deformations between shapes. The distances between shapes (or populations of shapes) can be computed within this space to quantitatively evaluate their difference.^119^ While originally developed in a context of medical imaging, the methods were applied to other biological domains as well.^62^, ^107^, ^120^

### 5.2 Filaments morphology

When the structure is a filament or a curve, the notions of area, thickness, or enclosed volume are no longer relevant, and different features have to be envisioned. To describe the morphology of a filament, the simplest feature is its length. Several features can be measured for different positions along the curve, such as the angle or the local curvature. When the curve was obtained as the medial axis of an elongated region, the thickness along the medial axis can be considered. For 3D curves, the variations of features measured on the 2D cross sections can also be considered.^81^ The average value of such features, or other summary statistics, can quantitatively describe the curve. The length can also be related to the spatial extent, using for example the Feret diameter, to obtain a measure of the tortuosity factor.

### 5.3 Networks morphology

The quantification of morphology for a network‐like structure is usually achieved by considering its branches, junction points, and eventually end‐points. To do so, the first step is to apply a classical segmentation procedure, followed by a ‘skeletonisation’ operation, that will compute a medial axis composed of all the points located at equal distance of at least two boundary points. See Saha et al.^121^ for an overview of skeletonisation algorithms. The topology of the network can then be described by using tools from graph theory: number of loops, distribution of the number of branches crossing at a given node, etc.^122^ For example, the number of branches during the development of a neural network reflects the abnormalities in development and disease.^123^ In ophthalmology and developmental studies of vascular systems, the vasculature network is described by a variety of approaches including fractal dimension, skeleton density, lacunarity analysis, or tortuosity.^91^, ^92^, ^124^ Similar approaches can be applied to the analysis of cytoskeletal networks^125^ or the endoplasmic reticulum.^97^, ^98^ Plant root branching is affected by the availability of resources such as water and soil, affording different scientific questions that can be addressed by similar analysis strategies.^126^, ^127^ Practical steps to analyse tumour blood vessel networks have been reported for the diagnosis of the progression of cancer.^128^

Furthermore, measurable features can be associated with nodes or edges, such as, the thickness or the length of the edges. Analysis can then be performed by extracting statistics for the different types of edges (e.g., branch length histogram). For example, average branch length of the network of connections between glial cells of zebrafish can be related to development.^129^ Topological features may also be analysed with respect to their location. For example, in neuron research, a classical analysis counts intersections of dendrites with concentric circles of increasing radius.^130^, ^131^ In plant sciences, a similar approach was applied to study the number of branching points of a fruit vasculature network with respect to the distance to the fruit centre.^99^

### 5.4 Porous media

When considering porous media, the quantification of morphology may be difficult to apprehend, as there is no obvious ‘region’ to relate to, and classical morphological features cannot be easily extracted. However, several approaches have been proposed to quantify the morphology of porous media, most of them originating from material sciences.^132^, ^133^, ^134^ A popular approach is to identify elementary components of either the solid or the void phase, and quantify their size distribution. The components may be the individual grains that form the structure, or the elementary voids or empty spaces between solid components. For example, the thickness of the elements can be related to the overall stiffness or solidity, or the diameter of the pores can be related to diffusion properties.

In case it is difficult to discretise the porous media into elements, a more global characterisation of the microstructures needs to be considered. The porosity, defined as the ratio of the void volume over the observed volume, is an intuitive feature that describes the density of the phase. Its counterpart is the volume fraction, defined as the ratio of the volume of the solid phase over the observed volume. Porosity or volume fraction may be related to the solidity of the material (e.g., bone),^68^ or to the circulation of gas within the void phase.^74^, ^135^ The specific surface, defined as the ratio of surface area over the observed volume, quantifies the ‘quantity of boundary’ and is of interest in the study of diffusion phenomena. Specific surface area was also found a key indicator in pulmonary disease quantification.^136^ The notion of connectivity used for analysing the percolation phenomenon, is often of specific interest.^137^ A related concept is the tortuosity, usually based on the computation of a geodesic distance.^137^, ^138^, ^139^ The notion of size distribution can also be addressed through the computation of local thickness. The principle typically consists in computing the skeleton of the solid phase, identifying the size of the largest disk or ball centred on each point of the skeleton, and computing the size distribution.^137^, ^140^ The same approach can be applied to describe the void phase, leading to different interpretations of the results. Stereological methods based on the covariance function or the pair‐correlation function can also provide a global quantification of the media.^141^, ^142^, ^143^ Other features have been proposed to quantify the variations of thickness with respect to the orientation.^144^, ^145^ Several software packages have been developed to facilitate the diffusion of such methods.^21^, ^146^, ^147^, ^148^, ^149^

## 6 CONTENT (INTENSITY, COLOUR, ETC.)

### 6.1 Regions

Regions are not defined only by their morphology, and the values of pixels within the regions may also be of interest. In the case of monochromatic images (i.e. grey levels or intensity images), the average intensity of pixels within the region can be used as an additional feature to describe the region. Other summary statistics such as the variance or the standard deviation can also be used to quantify homogeneity or heterogeneity of intensities within regions. The differences in summary statistics may be used to discriminate between regions with different classes (e.g., healthy vs. unhealthy cell), or to track the evolution of a physical phenomenon within the region.^13^, ^117^, ^118^ Beyond such simple statistical measures, more specific features can be obtained from the study of local variations of intensities or grey levels within each region. This is the aim of image texture analysis (see the corresponding section hereafter).

In the case of colour image or of multi‐channel images, each pixel is associated with several values. Summary features can be computed for each channel independently. The channels may be correlated, and dimensionality reduction techniques such as principal component analysis can help provide a synthetic view of the information. Other data types may provide more complex data types represented by a large number of values: fluorescence life‐time imaging, infrared or Raman micro spectroscopy, mass‐spectrometry imaging, etc. In these cases, the quantity of information requires adequate usage of multivariate image analysis methods.^150^, ^151^, ^152^, ^153^

### 6.2 Image texture analysis

The local variations of intensities of neighbour pixels also carry information that can be useful for analysis. When pixels of a certain area have similar or gradually changing intensities, they look smooth. On the other hand, when pixel intensities are abruptly alternating, they give the visual sensation that the surface is rough. This roughness can be different depending on the periodicity and randomness of intensities. Such visual impressions can be quantified by texture analysis. There are several approaches for this quantification.^154^, ^155^ The most popular one is the notion of grey‐level co‐occurrence matrices (GLCM), which computes the probabilities (‘co‐occurrence matrices’) that a pixel with a given value is at a distance from a pixel with another value.^154^, ^156^, ^157^ For example, the size of GLCM is 256 × 256 if the pixel value varies between 0 and 255. Using those probabilities, various texture feature values such as ‘contrast’, ‘dissimilarity’, and ‘homogeneity’ are computed as single values. Alternative approaches are the run‐length matrices that decompose images into elementary runs of consecutive pixels with constant or similar grey values,^158^ or the box counting fractal dimension, which measures how an image is sampled in ‘boxes’ of decreasing size^39^, ^40^ Image texture may also be investigated by applying image transforms beforehand to better identify relevant features. Examples of such preprocessing include Fourier transform, wavelets, or Gabor transform.^159^ Some techniques build predictive models of grey‐level variations, using for example autoregressive models, or Markov random fields, and use parameters of those models as texture descriptors. Within the domain of mathematical morphology, the grey‐level granulometry approach results in pattern spectrum curves that can be interpreted in the same way as classical size distributions.^160^, ^161^, ^162^

Image texture features may be computed at different scales. At the maximum scale, texture features are computed with the whole image. Instead, texture features can also be measured for specified regions. Note that for a reliable measurement of texture features, a sufficient number of neighbourhood pixels is required, so the size of such regions must be sufficiently large. At the pixel level, it is also possible to use the texture features computed around each pixel as input for pixel classification or segmentation approaches.^37^, ^38^, ^163^

## 7 DYNAMIC ANALYSIS

### 7.1 Tracking

Tracking determines the change in position of the target structure over time and is a widely used image analysis approach for measuring movements. In many cases, the shape of the target structure is diminished to a point by image processing, for example, centroid, representing the position of the structure.^164^, ^165^ This procedure is often called ‘detection’. These detected points are then linked over time using various algorithms, especially to avoid mistakes in linking different dots and to accommodate vanishing and newly appearing dots, to estimate movement trajectories. This process is called ‘linking’.^24^, ^166^, ^167^, ^168^, ^169^ In the case of near‐spherical objects such as intracellular vesicles, their detection resembles dot detection algorithms. For more complex shapes such as single cells, the geometric centroid is often used to represent the cell position (Figure 7).

Basic movement features such as trajectories, velocities, and directionalities are computed from tracking results. To avoid the bias of sampling interval on the estimated velocity, tracks can be analysed using mean‐square displacement (MSD) plots, which allows the estimation of the activities of random movement and biased (directed) movement separately.^170^ For small spherical structures, a well‐known diffusion equation can be curve‐fitted to the MSD plot, and the inclusion of the anomalous exponent, α, to the equation allows the estimation of the diffusive behaviour with steric hindrance or with active transport.^171^, ^172^, ^173^ For more complex shapes such as cells, Fuerth's formula, which incorporates the assumption of random movement intervened by persistent movement, is recommended for fitting MSD plots.^174^, ^175^, ^176^ Significant bias in the movement suggests a certain directionality of movement, and for deriving a proper statistical measure of directed movement, circular statistics is recommended.^177^, ^178^

Cell migration studies offer great examples of measuring the shape changes of regions as contour shape dynamics. A general approach for segmenting cell contour is intensity thresholding and binarisation.^166^, ^179^ A more robust approach less affected by intensity variations at the cell edge is active contour algorithms to automatically delineate cell contour for quantitative analysis of cell edge advancement and retractions.^180^, ^181^, ^182^

### 7.2 Kymographs

Time‐lapse imaging of filament structures results in large three‐dimensional data arrays containing a large amount of unnecessary data. A typical approach to analyse this type of data is to consider the kymograph, a two‐dimensional image that associates one axis to the position on the curve and the other axis to the time.^183^, ^184^, ^185^, ^186^ In this way, various dynamics, especially those that are difficult to segment can be measured for the speed of the movement, for example with proteins.^187^ In plants, the growth of hypocotyl and roots has been measured by reducing their shape to curvilinear regions using skeletonisation and by matching skeleton positions between successive frames to determine the zones of active growth and curvature formation.^188^, ^189^, ^190^, ^191^ Other applications include the dynamic instability of microtubules^192^, ^193^ or analysis of protein localisations within filopodia in relation to other features.^83^, ^84^

## 8 SPATIAL ORGANISATION

### 8.1 Regions

In addition to the morphology or the intensity features, the spatial organisation of the regions is also of particular interest. Indeed, the regions are often interpreted depending on their relative location to a reference region: the boundary of the enclosing cell or organ, its centre, a specific anatomical point, etc. Regions can be at the periphery, in the centre, close to an extremity, etc. This kind of spatial relationship can be addressed by computing the distance between the regions (or of their centroid) with the reference structure, and interpreting regions features according to this distance. For example, distances of gene loci to nuclear envelope was measured to evaluate the level of the expression of each gene.^194^ It is also possible to analyse regions depending on whether they are included within the reference region(s), in a ‘parent‐child’ relationship.^195^ The ‘mereotopology’ approach, originally developed to enhance segmentation, aims at describing more formally how several regions are organised together, for example, A is on the left of B, B is within C, etc.^196^, ^197^, ^198^

When analysing images of plant tissues composed of many cells, one can also consider a topological distance, by counting the number of cells one has to travel through in order to reach the reference region. This type of analysis requires the construction of a region adjacency graph (RAG) between the regions, where the nodes of the graph correspond to the regions, and the edges denote a pair of adjacent regions. The use of a region adjacency graph allows for using graph‐theory methods, such as the computation of shortest paths between relevant regions. Dedicated software tools have been developed for the construction and analysis of such graphs.^199^, ^200^ Physical information may be associated with nodes or edges of the graph. For example, cells exchange information via chemical signals and physical stresses that can be associated with the corresponding edges.

The adjacency between regions is also useful to analyse the features of a region in relation to those of its neighbours. Indeed, cells may be associated to a specific tissue or cell layer, such as the epidermis, or to groups of cells arising from successive divisions.^201^, ^202^, ^203^, ^204^ A typical question is the identification of groups of neighbour regions that share some features.^205^

### 8.2 Filaments

In the case of filaments (and flat regions in 3D), another key feature for describing their spatial organisation is their orientation. Orientation can be computed for a collection of regions, or in relation to another structure used as a reference.^67^ Orientation can also be quantified locally, by considering the local neighbourhood of each pixel to detect the directionality of intensity pattern in that region.^206^ Note that when considering patterns of thin and parallel structures, the tools from image texture analysis may also be effective.

### 8.3 Point patterns

When considering point patterns, different questions may arise: What is the number (or the numerical density) of the points? Are there regions within the image where the points are more numerous? Do the points interact with each other and form clusters, or on the contrary do they tend to repulse each other? Methods from **spatial statistics** have been developed to describe quantitatively point patterns regardless of what those points represent: position of trees, cell centroids, stars in a galaxy, etc.^207^, ^208^ Comprehensive introductions to the analysis of biological images in terms of point patterns were proposed.^209^, ^210^ The features to quantify omit the size or the shape, but focus on the notions of density and spatial organisation. This family of methods has recently increased in interest in the context of Single‐Molecule Localisation Microscopy (SMLM),^46^, ^211^, ^212^, ^213^, ^214^ and with the rise of spatial omics.^200^, ^215^, ^216^, ^217^

**FIGURE 9:**
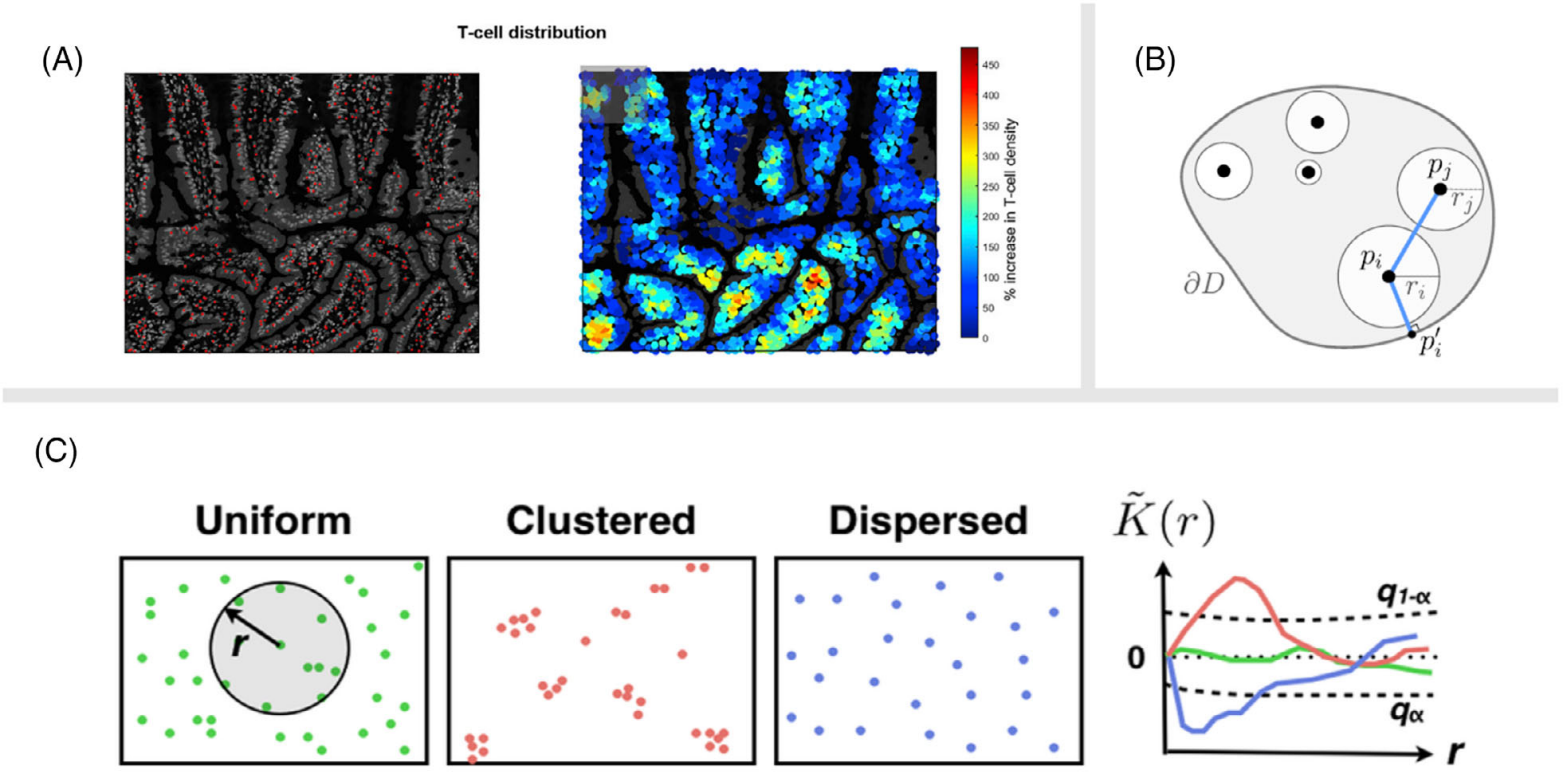
Illustration of quantitative analysis methods for point patterns. (A) Computation of density maps may help reveal visually the regions where a specific cell type is more present.^210^ (B) The position of points may be related to each other, or to a reference structure such as the boundary of the enclosing region.^220^ (C) Summary functions such as the K‐function can discriminate from random distribution, clustering, or repulsion.^49^

The first way to describe point patterns is to use a density map. In a density map, each pixel of the map has a value that corresponds to the expected number of points in the neighbourhood. Density maps can be computed by applying signal processing tools such as Parzen filters, or by defining the density from the distance to the *k*th nearest point (Figure 9A). Density maps can then be investigated with image analysis tools. For example, organisation of biological component densities can be studied with respect to the belonging biological structure (Figure 9B).^48^, ^218^, ^219^

Point patterns may also exhibit spatial interactions, such as attraction or repulsion. Tools from spatial statistics are of high interest to quantify such interactions. A family of methods employ summary functions that quantify the degree of interaction depending on a given distance (Figure 6C). The most common one of such is the ‘K‐function’, introduced by Ripley, which counts the expected number of points within a series of disks with increasing radius r centred on a typical point.^221^ The result of this measurement is a varying density plotted against distance from each point. Deviations of this plot from a quadratic curve denote an aggregation (clustering) or on the contrary a repulsion effect. Several variants have been proposed (the L‐function, H‐function, F‐function, etc.) to facilitate the interpretation.^49^ Note that the result of the analysis is not a single scalar value such as the number of points, but a function, thus requiring further investigations and possibly analyses to draw a conclusion.

In the case of imaging of multiple proteins tagged with probes with different emission spectra, resulting in point patterns in two different channels, a common question is to quantify the colocalisation of these two patterns. A large number of object‐based colocalisation analysis methods have been proposed to quantify the colocalisation of the two families of proteins, based on the overlap of the dots.^222^, ^223^

As a complement of quantification from summary functions, the domain of stochastic geometry has proposed various models to represent geometric patterns. In a similar way random values can be generated using a pre‐defined number distribution (Normal, Poisson, etc.), point pattern models can be used to randomly generate synthetic point patterns with organisation similar to that of the observations.^224^, ^225^, ^226^, ^227^

## 9 CONCLUSIONS

In conclusion, advancing the field of bioimaging necessitates fostering a collaborative environment where biologists and image analysts share a common language and understanding. Our study highlights the importance of simplifying the bridge between complex bioimage datasets and the extraction of meaningful biological data. By categorising biological structures and linking them to appropriate analysis methods, we provide a valuable resource for early career scientists and researchers. This approach not only aids in selecting suitable analysis techniques but also fosters better communication between biologists and image analysts. Our flowchart serves as a practical tool, simplifying the complex process of bioimage analysis and enhancing the ability to extract meaningful insights from biological data. Ultimately, this work aims to streamline bioimage analysis workflows, making advanced techniques more approachable and effective for diverse research applications.
